# Identifying medical phenotypes associated with microglia‐expressing AD risk genes

**DOI:** 10.1002/alz.093573

**Published:** 2025-01-03

**Authors:** Xue Zhong, Dan Zhou, Bingshan Li, Nancy J Cox

**Affiliations:** ^1^ Vanderbilt Genetics Institute, Vanderbilt University Medical Center, Nashville, TN USA; ^2^ Vanderbilt University, Nashville, TN USA

## Abstract

**Background:**

A recent study with large samples of electronic health records (EHRs) suggested Shingles vaccination may reduce dementia risk. Although further investigation is needed to pinpoint the underlying mechanism, such observation adds to the evidence for a connection between peripheral and central nervous system immunity. Since microglia is the major cell type implicated in AD genetics, here, we set out to probe the shared biology between microglia in human brain and macrophages in peripheral system, through the common genes that express in both cell types.

**Method:**

First, we identified a set of high‐confidence AD risk genes through integrative analysis of AD GWAS and human‐brain‐related genomic features. Then, we used eQTLs from human microglia to derive the eGene direction affected by AD risk alleles. For these AD‐associated eGenes, we predicted their expressions in peripheral tissues (whole blood, gut, lung, liver etc.) known to be enriched for macrophages with the use of UK biobank (UKB) data and prediction models pre‐trained by Genotype‐Expression project (GTEx) data. Finally, we tested the association of these genetically‐predicted gene expressions across ∼1000 medical phenotypes in UKB., followed by a replication analysis in the biobank of Vanderbilt University Medical Center (VUMC) to identify robust gene‐phenotype associations.

**Result:**

The resulting associations (after adjustment for multiple testing), implicated inflammatory phenotypes, which include the association of connective tissue diseases with increased expression of CTSH, Emphysema with increased expression of TSPAN14, Edema with increased expression of PTK2B, Herpes simplex with reduced expression of PLCG2, and uterine inflammatory disease with increased expression of CD79B. The directions of these associations are all consistent with the regulatory direction in gene expression in human microglia by AD risk alleles.

**Conclusion:**

We demonstrated that in peripheral tissues enriched for macrophages, AD risk genes that function in microglia were associated with inflammatory phenotypes in directions consistent with that observed in microglia, suggesting shared immune biology.

